# Attributes Underlying Patient Choice for Telerehabilitation Treatment: A Mixed-Methods Systematic Review to Support a Discrete Choice Experiment Study Design

**DOI:** 10.34172/ijhpm.2021.150

**Published:** 2021-11-03

**Authors:** Lucien P. Coulibaly, Thomas G. Poder, Michel Tousignant

**Affiliations:** ^1^Université de Sherbrooke, Sherbrooke, QC, Canada.; ^2^Centre de Recherche sur le Vieillissement, Sherbrooke, QC, Canada.; ^3^Département de Gestion, Évaluation et Politique de Santé, École de santé publique de l'Université de Montréal, Montréal, QC, Canada.; ^4^Centre de recherche de l'Institut universitaire en santé mentale de Montréal, Montréal, QC, Canada.

**Keywords:** Telerehabilitation, Preference, Choice, Discrete Choice Experiment, Satisfaction

## Abstract

**Background:** Across most healthcare systems, patients are the primary focus. Patient involvements enhance their adherence to treatment, which in return, influences their health. The objective of this study was to determine the characteristics (ie, attributes) and associated levels (ie, values of the characteristics) that are the most important for patients regarding telerehabilitation (TR) healthcare to support a future discrete choice experiment (DCE) study design.

**Methods:** A mixed-methods systematic review was conducted from January 2005 to the end of July 2020 and the search strategy was applied to five different databases. The initial selection of articles that met the eligibility criteria was independently made by one researcher, two researchers verified the accuracy of the extracted data, and all researchers discussed about relevant variables to include. Reporting of this systematic review followed the Preferred Reporting Items for Systematic Reviews and Meta-Analyses (PRISMA) guidelines and the Mixed Methods Appraisal Tool (MMAT) was used to assess the quality of the study. A qualitative synthesis was used to summarize findings.

**Results:** From a total of 928 articles, 11 (qualitative [n = 5], quantitative [n = 3] and mixed-methods [n = 3] design) were included, and 25 attributes were identified and grouped into 13 categories: Accessibility, Distance, Interaction, Technology experience, Treatment mode, Treatment location, Physician contact mode, Physician contact frequency, Cost, Confidence, Ease of use, Feeling safer, and Training session. The attributes levels varied from two to five. The DCE studies identified showed the main stages to undertake these types of studies.

**Conclusion:** This study could guide the development of interview grid for individual interviews and focus groups to support a DCE study design in the TR field. By understanding the characteristics that enhance patients’ preferences, healthcare providers can create or improve TR programs that provide high-quality and accessible care. Future research via a DCE is needed to determine the relative importance of the attributes.

## Introduction

 The concept of patient-centered care has received increased attention in recent years and is considered an important goal for healthcare system improvement.^1^ Healthcare services are increasingly moving away from a paternalistic approach, ie, doctor-knows-best method, toward a way where patients play more active roles in their care.^2^ Across most healthcare systems, patients are the primary focus and there has been increased calls for their involvement in healthcare decision-making.^3,4^ Patient involvement has been shown to enhance patients’ adherence to treatment, which in return, influences their health.^5^ Furthermore, other benefits of patient involvement are notably improvements in healthcare policies, shared decision making, and taking into consideration patients’ preference while also accounting for their changing health state.^6^ It is important to know and consider what drives patients’ choice for treatment and to better understand their preferences for the various attributes (ie, characteristics) within a given treatment.^7^ There might be several underlying preferences that explain patients’ choice. While choice modelling is used to better understand how choices are made, by quantifying the strength of underlying preferences, and to forecast future choice responses,^8^ discrete choice experiment (DCE) is a survey-based stated-preference elicitation technique consisting of a series of hypothetical choice situations (called choice sets), each concerning a discrete choice between two or more alternatives. The presented alternatives in a DCE are decomposed into characteristics (called attributes) to describe the alternatives that are distinguished from one-another by the systematic variation in the values of the characteristics (called attributes’ levels).^8^ To identify attributes and their associated levels regarding patients’ choice is crucial for a successful DCE study design.

 For outpatients needing rehabilitation services at home or elsewhere by telerehabilitation (TR), considering their preferences appears to raise interest on their involvement and to focus on their satisfaction, which could lead to better health outcomes. TR is defined as “The use of Information and Communication Technologies to provide rehabilitation services to people remotely in their homes or other environments.”^9^ TR is a new approach in the rehabilitation field and is considered safe^10,11^ and effective,^11-13^ allows to reduce costs^14,15^ (mostly with transportation) and improve quality of life^16^ when compared to face-to-face rehabilitation. This service has been identified as a very promising alternative tool that could help to offer and improve early access to healthcare services, and patients using that service reported high levels of satisfaction.^17,18^

 Much of the published studies on TR has been limited to patients’ satisfaction, perceptions, or experiences after they have used the service.^14,19,20^ The findings generally reveal these factors (eg, patients’ satisfaction level) without a focus on the attributes and levels of TR.^21^ Yet, to better inform patient-centered TR design, it is important to know and understand which attributes are the most important to patients in their process to choose or not TR.^17^ Methods used to obtain attributes could include: systematic review; theoretical arguments from the literature; grey literature; professional recommendations; qualitative research methods (eg, in-depth interviews, focus groups); patient surveys; and expert review.^22^ To our knowledge no mixed-methods review (ie, literature review that concomitantly examines qualitative, quantitative and mixed methods primary studies)^23^ were conducted to support the qualitative step of a DCE study design in the field of TR. To date, the few DCEs studies in the field of TR did not conduct systematic review prior to the qualitative step.^17,24^

 To know more about the most important attributes of TR and to better understand the preferences of patients influencing the choice of TR, it is important to conduct a mixed-methods systematic review to generate a potential list of attributes before the qualitative phase. This list is not meant to be exhaustive, rather, it will guide the development of tools to support the qualitative phase of a DCE design in TR field.^25^ The objective of this mixed-methods systematic review is therefore to determine the attributes and their associated levels that are the most important for patients regarding TR healthcare to support the qualitative step of a future DCE study design.

###  Research Question

 The review sought to answer the following question: “What are the main attributes and their associated levels that are the most important for patients regarding TR healthcare?”

## Methods

 We conducted this systematic review according to the Preferred Reporting Items for Systematic Reviews and Meta-Analyses (PRISMA) Statement.^26^ The checklist depicting how this review aligns with the PRISMA approach is presented in Supplementary file 1. The protocol for this systematic review was not registered in the international database of Prospectively registered systematic reviews in health and social care (PROSPERO). However, the internal protocol is available upon request from the corresponding author. A qualitative synthesis of the available evidence (ie, data extracted) was carried out into an Excel spreadsheet.

###  Search Strategy

 Studies were identified in five different electronic bibliographic databases: (1) PubMed, (2) MEDLINE via EBSCO, (3) ScienceDirect, (4) Cochrane Library and (5) Scopus. In searching, the search concepts, and key words such as: “telerehabilitation”; “preference”; “choice”; “discrete choice experiment”; “treatment choice”; “satisfaction”; “perception”; “adherence”; “acceptance” were combined, using Boolean operators “OR” and “AND.” The complete keywords and search strategies used are available in Supplementary file 2. To ensure that all the potentially relevant articles were identified, references from the lists of retrieved articles were screened to identify additional potentially relevant articles.

###  Eligibility Criteria

 To be included, articles needed to: (1) be published in English or French; (2) involve patients with health conditions requiring TR (eg, teleconsultation, telemonitoring, and teletreatment) regardless the time period and number of interventions; (3) include an economic evaluation of TR (ie, assessed the impact of the use of TR on the costs for patients); (4) determine the preference, adherence, satisfaction, and perception of TR from patients; (5) include studies in the field of TR analyzing health preferences that used mixed-methods design and methods (eg, DCE design that include the fourth main steps, ie, development of attributes and attribute-levels, development of DCE survey and choice sets, administration of DCE survey and statistical and econometric analysis); (6) use quantitative design that include experimental, quasi experimental or non-experimental study design; (7) use qualitative methods for data collection and data analysis; and (8) be published from 2005 to end of July 2020 because we considered the most recent publications to be more relevant to the current research efforts. Studies that did not meet at least one of inclusion criteria 5 or 6 or 7 were excluded.

###  Outcomes

 In this review, outcomes refer to the main attributes and their associated levels that are the most important for patients regarding TR healthcare. Outcomes that will be considered are those that will assist in addressing the research question.

###  Study Selection Process

 The initial selection of articles was independently made by one researcher (LPC) who performed the following tasks: screening titles and abstract and full-text selection of the potentially eligible studies identified in the database searches. Two other researchers (TGP and MT) verified the search strategy and validated the inclusion or exclusion of articles. If required, disagreements were solved with a general discussion performed by every three researchers (LPC, TGP, MT).

###  Data Extraction Process

 One researcher (LPC) extracted the variables of interest from each of the included studies: authors, year, country, study design, methods for data collection, methods for data analysis, population, number of participants, eliciting preferences stages, methods to estimate relative attributes importance, number of choice sets per respondent, attributes, levels, quality assessment (Mixed Methods Appraisal Tool [MMAT] Score). Two researchers (TGP and MT) verified the accuracy of the extracted data. In case of discrepancy, consensus was reached through discussion between all researchers (LPC, TGP and MT), and they discussed the addition or subtraction of certain variables of interest.

###  Data Analysis

 We provided descriptive statistics on the characteristics of the reviewed studies. A qualitative synthesis of the available evidence was conducted. This process of data analysis and synthesis consisted of 3 steps: (1) Qualitative data of each included articles were extracted and entered an Excel spreadsheet; (2) Major/recurrent attributes were identified and refined into key “attributes”; (3) The common attributes to studies were summarized under thematic headings representing the major attributes and their levels.

###  Quality Assessment

 To assess the quality of included studies, the MMAT was used.^27^ This tool contains five specific sets of criteria: (1) a ‘qualitative’ set for qualitative studies, and qualitative components of mixed methods research; (2) a ‘randomized controlled’ set for randomized controlled quantitative studies, and randomized controlled components of mixed methods research; (3) a ‘non-randomized’ set for non-randomized quantitative studies, and non-randomized components of mixed methods research, (4) an ‘observational descriptive’ set for observational descriptive quantitative studies, and observational descriptive components of mixed methods research; and (5) a set ‘mixed methods’ for mixed methods research studies.^28^ Each study type is judged within its methodological domain. To the best of our knowledge, it is the only tool that allows to concomitantly appraise the methodological quality of quantitative, qualitative, and mixed-methods studies, using a valid and usable specific set of criteria studies.^29^

## Results

###  Selection of Studies

 The PRISMA flowchart in Figure details the approach of our research strategy and the flow of articles through the study. We identified 928 records through the searches and 12 additional articles were identified by searching through other sources, which brought the total of potentially relevant articles selected for analysis to 940 (see Figure). After removing duplicates and reviewing the titles and abstracts, 43 articles were identified for full reading. In the 43 articles selected for full reading, 32 of these were excluded (Supplementary file 3) based on the pre-specified inclusion and exclusion criteria.

**Figure:**
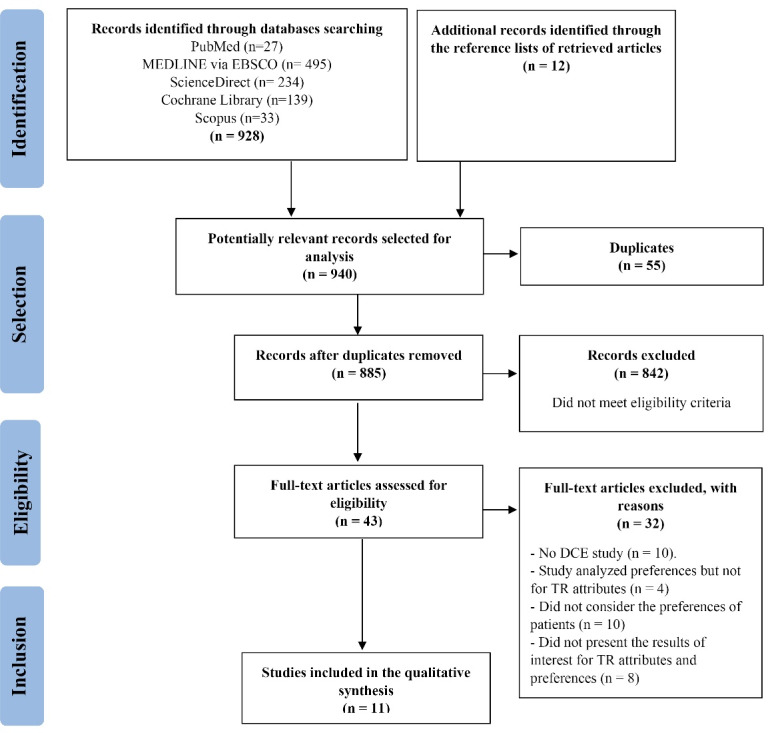
PRISMA Flow Diagram on End of July 2020. Abbreviations: PRISMA, Preferred Reporting Items for Systematic Reviews and Meta-Analyses; TR, telerehabilitation; DCE, discrete choice experiment.

###  Studies’ Characteristics 

 Of the 11 studies, three were conducted in Australia, two were conducted both in Canada and Netherlands and one in each of the following countries: Italy, Norway, USA, and Singapore. These articles were published between 2013 and 2019. Study designs varied, but most studies were qualitative (n = 5),^30-34^ quantitative (n = 3),^14,16,35^ and mixed-methods designs (n = 3).^17,24,36^ Between mixed-methods’ studies, two were DCE designs^17,24^ and their qualitative component included respectively 61 (ie, 13 patients, three spouses and one carer [interviews], and 44 healthcare and allied services professionals [focus groups]^24^) and 16 participants (ie, 10 patients [interviews] and six healthcare professionals [focus groups]^17^). Concerning the quantitative stage, respectively 330^24^ and 300 participants^17^ were involved. Nine articles^14,30,31,35-40^ were about patients’ satisfaction, perception, and adherence. In these studies, sample sizes varied from 10 to 102 participants. According to the MMAT methodological quality criteria,^23^ all quantitative studies^14,16,35^ and most of the qualitative studies^31-34^ were of average quality except one qualitative study^30^ that was of low quality. All mixed-method studies were of high quality with common strengths including (1) the combination of at least one qualitative method and one quantitative method; and (2) each method was used rigorously in accordance with the generally accepted criteria in research invoked. Tables 1, 2, 3 and 4 summarize the characteristics of the included articles.

**Table 1:** Characteristics of Discrete Choice Experiment Studies

**Authors (Year of Publication)**	**Country**	**Study Design**	**Methods for Data Collection**	**Methods to Estimate Relative Attributes’ Importance**	**Number of Choice Sets Per Respondent**	**Population**	**Number of Participants (Patients and Stakeholders)**	**Eliciting Preferences Stages**	**Quality Assessment (MMAT Score)**
Kaambwa et al (2016)	Australia	Mixed methods	DCE questionnaire Interviews + focus group	Mixed logit model Thematic analysis	6 choice sets	Older people aged 65 years	DCE (n = 330 patients) Interviews (n = 17, ie, 13 older patients, three spouses and one carer) Focus group (n = 44, ie, healthcare, and allied services professionals)	(1) Development of attributes and attribute-levels (2) Development of DCE survey and choice sets (3) Administration of DCE survey (4) Statistical and econometric analysis	****
Cranen et al (2017)	The Netherlands	Mixed methods	DCE questionnaire Interviews + focus group	Bivariate probit model Not defined	15 choice sets	Patients with chronic pain	DCE (n = 300 patients) Interviews (n = 10 patients) Focus group (n = 6 healthcare professionals)	(1) Identification of the key treatment attributes and assignment of levels to the attributes (2) Design of the experiment and determination of hypothetical treatment scenarios using various combinations of attributes and levels (3) Choosing an elicitation format and obtaining choice data in patients (4) Analysis of the choice data	****

Abbreviations: DCE, discrete choice experiment; MMAT, Mixed Methods Appraisal Tool. Notes: MMAT quality assessment categories range from * (lowest) to **** (highest).

**Table 2:** Attributes and Levels of Discrete Choice Experiment Studies

**Authors (Year of Publication)**	**Attributes**	**Levels**
Kaambwa et al (2016)	Aspects of care in telehealth sessions	All aspects of care Most aspects of care A few aspects of care
	Distance to nearest hospital or clinic	Less than 15 km away Between 15 and 100 km More than 100 km
	Clinicians’ attitude to telehealth	Very positive Moderately positive Uncertain
	Technology-experience levels of patients targeted by telehealth	A lot of experience Some experience No experience
	Assessments related to telehealth sessions	All assessments Some assessments No assessments
	Cost of telehealth	$0 $40 $80
Cranen et al (2017)	Treatment mode and location	You exercise in a group at the gym You exercise individually at the gym You exercise individually at home You exercise in a virtual group at home
	Physician contact mode	All physician contact takes place at the clinic face-to-face One quarter of your physician contact through Web camera Three-quarters of your physician contact through Web camera All your physician contact takes place through Web camera
	Physician contact frequency	Every exercise session you will have physician consulting Once per 2 exercise sessions you will have physician consulting Once per 3 exercise sessions you will have physician consulting Once per 4 exercise sessions you will have physician consulting
	Feedback and monitoring technology	Use of technology—feedback and monitoring of your exercises No technology—feedback and monitoring of your exercises
	Program flexibility	Fixed exercise times Flexible exercise times
	Healthcare premium reduction	No discount €50 discount €150 discount €450 discount

**Table 3:** Characteristics of Satisfaction, Perceptions, and Adherences’ Studies

**Authors (Year of Publication)**	**Country**	**Study Design**	**Methods for Data Collection**	**Methods for Data Analysis**	**Population**	**No. of Patients**	**Quality Assessment (MMAT Score)**
Bedra et al (2013)	USA	Qualitative	Semi-structured interviews	Not defined	Patients with chronic obstructive pulmonary disease	n = 21	*
Crotty et al (2014)	Australia	Qualitative	Interviews	Narrative presentation	Patients who needed rehabilitation (eg, following a stroke, a fracture or prolonged hospital admission)	n = 78	***
Paneroni et al (2014)	Italy	Quantitative	Questionnaire	Statistical analysis	Patients with chronic obstructive pulmonary disease	n = 36	***
Marquis et al (2015)	Canada	Quantitative	Questionnaire	Statistical analysis	Patients with chronic obstructive pulmonary disease	n = 26	***
Edgar et al (2017)	Canada	Quantitative	Questionnaire	Statistical analysis	Stroke survivors	n = 102	***
Hoaas et al (2016)	Norway	Mixed methods	Focus groups Individual open-ended questionnaire	Thematic analysis Narrative presentation Statistical analysis	Patients with chronic obstructive pulmonary disease	n = 10	****
Brouns et al (2018)	The Netherlands	Qualitative	Focus groups	Content analysis	Stroke patients	n = 60	***
Tyagi et al (2018)	Singapore	Qualitative	Focus groups In-depth interviews	Thematic analysis	Stroke patients	n = 37	***
Wentink et al (2019)	The Netherlands	Qualitative	Focus groups	Content analysis Narrative presentation	Stroke patients	n = 47	***

Notes: MMAT quality assessment categories range from * (lowest) to **** (highest).

**Table 4:** Attributes and Levels of Satisfaction, Perceptions, and Adherences’ Studies

**Authors (Year of Publication)**	**Attributes**	**Levels**
Bedra et al (2013)	Feeling safer	Significantly safer Moderately safer
	Usability	Not Complicated at all Slightly Complicated
Crotty et al (2014)	Technology experience	Never Rarely or more than once a year More than once a month More than once a week More than once a day
	Usability Scale after exposure to TR	1 = strongly disagree to 5 = strongly agree
Paneroni et al (2014)	Ease of use of the technology	Very easy Easy Not so easy Not at all easy
	Healthcare expectations	Very much Somewhat Little Not at all
	Training sessions	Not reported
Marquis et al (2015)	Training sessions	Not reported
Edgar et al (2017)	Accessibility	Not reported
	Location	Not reported
	Distance	Not reported
	Previous experience with technologies	Never Once per month Once per week Daily
	Confidence	Not confident Somewhat confident Very confident
Hoaas et al (2016)	Emotional safety	Not reported
	Training session	Not reported
Brouns et al (2018)	Accessibility	Not reported
	Feasibility	Not reported
	Privacy	Not reported
	Safety of patient data	Not reported
	Time	Not reported
	Financial arrangements	Not reported
Tyagi et al (2018)	Accessibility	Not reported
	Affordability	Not reported
	Interaction	Not reported
	Scope of exercises training session	Not reported
Wentink et al (2019)	Accessibility	Not reported
	Usability	Not reported

Abbreviation: TR, telerehabilitation.

###  Population Characteristics 

 All articles described the study population. Most participants were patients with strokes (n = 4),^14,31,36,40^ and with chronic obstructive pulmonary disease (n = 4).^30,35,38,39^ Other studies specifically involved patients with chronic pain (n = 1),^17^ patients with various diseases that needed rehabilitation (n = 1),^37^ and older patients (n = 1).^24^

###  Findings Related to Discrete Choice Experiment Studies

####  Identification of Key Attributes and Assignment of Levels

 Results from the DCE studies (n = 2) are reported in Tables 1 and 2. Qualitative interviews with patients and expert focus groups methods were used to select the key attributes and assign the levels.^17,24^ Kaambwa et al identified six salient attributes of telehealth: (1) aspects of care, (2) distance, (3) clinicians’ attitude, (4) patients’ experience with technology, (5) types of assessments, and (6) costs associated.^24^ Each of these attributes had three levels. The results highlighted, in order of the strength of preference, that patients favored telehealth services when: (1) clinicians were very positive or moderately positive about the telehealth service, (2) a clinician pursued all or most aspects of care during a telehealth session, (3) all or some of the health assessments took place in a clinic prior to a telehealth session, (4) targeted at those for whom the nearest hospital or clinic that could serve as an alternative to telehealth services was between 15 and 100 km away from their home, (5) targeted at individuals with some experience of using technology and, (6) a low cost was associated.^24^ In Cranen et al study, six treatments attributes were also identified, and the levels varied from two to four. In this study, the main attributes were: (1) treatment mode and location, (2) physician contact mode, (3) physician contact frequency, (4) feedback and monitoring technology, (5) program flexibility, and (6) healthcare premium reduction.^17^ Between these attributes, the physician communication mode, the use of feedback and monitoring technology, and exercise location were key drivers of patients’ treatment preferences (*P*< .001).^17^ The treatment scenario consisting of attributes associated with both conventional rehabilitation and TR was the most preferred. This means that patients were offered a clinical exercise environment with feedback and monitoring technology where face-to-face consulting with a physician was limited.^17^ In summary, the DCE studies allowed to identify 12 attributes, but three of them (aspects of care, clinicians’ attitude, and healthcare premium reduction) were common to both studies. Finally, these attributes can be grouped into nine categories: (1) distance, (2) physician contact mode, (3) patients’ experience with technology, (4) types of assessments, (5) costs associated, (6) treatment mode and location, (7) physician contact frequency, (8) feedback and monitoring technology, (9) program flexibility.

####  Developing the DCE Questionnaire, Choice of Scenarios and Data Analysis

 Attributes and their levels from the thematic analysis of focus groups and interviews were combined to produce hypothetical options and assigned to choice sets using experimental designs.^24^ Within a questionnaire, the respondents were presented with a sequence of choice sets made up of two or more competing alternatives that vary according to value of attributes’ levels. A commonly D-optimal experimental design algorithm was employed to reduce the number of choice sets to the smallest number required to generate statistically efficient preference estimates for the attributes and levels included.^17,24^ Sawtooth software^17^ and Ngene software^24^ were used to design the choice tasks. The relative importance of attributes was estimated using a bivariate probit regression analysis^17^ and a mixed logit model,^24^ respectively.

###  Findings Related to Satisfaction, Perception, and Adherence Studies

####  Attributes and Levels 

 Results of these studies (n = 9) are reported in Tables 3 and 4. The authors reported several attributes grouped into 15 main categories: (1) Accessibility,^14,31,36,40,41^ (2) Distance,^14^ (3) Interaction,^40^ 4) Financial arrangements,^36^ (5) Location,^14^ (6) Training sessions,^35,38-40^ (7) Time,^36^ (8) Feeling safer,^30,36,39^ (9) Confidence,^14,36^ (10) Technology experience,^14,37^ (11) Usability,^30,31,37^ (12) Feasibility,^36^ (13) Affordability,^40^ (14) Ease of use,^38^ and (15) Healthcare expectations.^38^ The Attributes’ levels varied from two to five. Nine of the 15 attributes reported did not have their level reported (Accessibility, Distance, Interaction, Financial arrangements, Location, Training session, Time, Feasibility, and Affordability).

 In summary, all attributes identified through the DCE studies and satisfaction, perception and adherence studies can be grouped in 25 attributes (ie, nine plus 16 categories). For common attributes to both kind of studies only one was kept. For instance, between three kinds of attributes, ie, “Cost of telehealth,”^24^ “Healthcare premium reduction”^17^ and “Financial arrangements,”^32^ only the “Cost” (as a generic term) was retained as a common attribute.

## Discussion

 We conducted a literature review that generated a comprehensive list of attributes and their levels that were the most important for the patients in their decision-making in the choice of a TR treatment. Overall, we identified 11 studies and 25 attributes. After keeping only one repeated attribute in both kind of studies, the attributes can be grouped into 13 categories: (1) Accessibility, (2) Distance, (3) Interaction, (4) Technology experience, (5) Treatment mode, (6) Treatment location, (7) Physician contact mode, (8) Physician contact frequency, (9) Cost, (10) Confidence, (11) Ease of use, (12) Feeling safer, and (13) Training session. The attributes levels identified through the included studies varied from two to five. The DCE studies identified showed the main stages to undertake these types of studies.

###  Attribute Development

 A good DCE study is one that has a sufficiently rich set of attributes, together with enough variation in the attribute levels necessary to produce meaningful responses from each respondent.^8^ It is highly recommended that qualitative research methods, that are a popular approach for identifying attributes and levels^6^ in preference studies, are conducted for developing DCE attributes. To include key stakeholders in the qualitative phase could help to develop more representative DCE attributes^24^ but could be also a limitation. In fact, the range of attributes and levels may not be truly inductive from the patient preference and satisfaction perspective. In line with recent methodological recommendations, the starting pointing in attributes and levels selection process should be a rigorous literature review on the topic.^22,38^ This first stage generates a potential list of attributes and their levels for inclusion. The systematic review synthesizes attributes and their levels of previous similar studies from both published and grey literature. Conducting a systematic review prior to the qualitative component of a DCE design is very important because the attributes list and their levels will guide the development of data collection tools (eg, interview grid and topic guides) to be used in the focus groups and individual interviews.^25,42^ Leading focus groups are becoming increasingly popular to inform about credible attribute levels, possible interaction effects and, more generally, to shed some light on the best way to introduce and explain the task to respondents.^8^

 However, the DCE studies identified in our review did not conduct a systematic review before the qualitative stage. The authors did not explain why this stage was omitted. This can be explained by several reasons: (1) there are no hard and fast rules used to determine the attributes and levels presented to respondents in a DCE^8,38,43^; (2) identified attributes and their levels from systematic review may be repetitive not relevant and not necessary^42^; and (3) a systematic review could generates a large list of attributes that increase the number of possible choice sets. These reasons are coherent with Whichello et al recommendations who suggested that the ideal number of attributes for patient preferences information elicitation studies should be between three and six.^44^ A higher number of attributes could lead to a larger number of alternatives and choice sets. This could negatively impact the response rate and can lead to patients’ fatigue.^45,46^ For example, for studies with more than seven attributes, the number of responses presented to respondents increase considerably and, the cognitive burden can become unmanageable for them.^14^ Although these reasons could support the authors’ choice, in line with the International Society for Pharmacoeconomics and Outcomes Research Good Research Practices for Conjoint Analysis Task Force,^38^ a systematic review combined with a qualitative phase allows to generate a list of comprehensive attributes to support a DCE study design. This would avoid a misspecification of the attributes and attribute-levels that could have great negative implications for the design and implementation of DCEs and a risk of producing erroneous DCE results which can misinform policy implementation.^22,42^

###  Attributes’ Heterogeneity

 Various attributes were observed in the included studies. The heterogeneity between the different attributes seems to be explained by the patients’ specific characteristics and the type of TR services that they receive. Individual characteristics such as living arrangements and presence or absence of a long-term disability may or not influence individuals’ preferences for attributes of telehealth service delivery.^24^ For example, stroke patients with significant rehabilitation problems tend to prefer doing exercises individually, at home in virtual, under the physicians’ guidance. In contrast, patients with chronic pain problems on the waiting list needing monitoring of their condition could prefer exercising in a virtual group at home, or some pre-recorded exercises to do when convenient. To prepare a DCE study about TR, including the most frequently cited attributes about TR from literature is essential because attributes redundancy from several sources could mean that it is important for patients. Although this list of attributes is not meant to be exhaustive; it could guide the development of interview grid and topic guides for individual interviews and focus groups. Although some attributes were mentioned in several studies, that is not a guarantee that these attributes are considered important for patients.^7^ As such, a DCE study is needed to determine the relative importance of the attributes for the patients, which may help in the design of TR interventions. There are other ways of underlying preferences. For example, “revealed preference” are seen as the gold standard, but data from this approach are scarce because there are difficulties getting them in a real-world context.^47^ This approach is less cognitively demanding for patients because data collection is performed in real-time during or just after the intervention or treatment unlike the DCE that consists of a series of hypothetical choice sets. Other factors could influence the revealed preference, hence, change what a patient would want. For instead, the context that includes pathway-related factors (eg, patient relocation, length and timing of the appointment) and symptom-related factors (eg, patient symptoms and the effect of travel on these).^48^ In the case of TR, the DCE stays the most appropriate method for collecting data on patients’ preferences because TR is a new approach in the rehabilitation field, consequently few data are available on patients’ revealed preference.

###  Attributes’ Levels 

 Levels need to be assigned to attributes and these may be cardinal, ordinal or categorical, or with no natural ordering.^43^ It is possible that various levels of TR could affect the preference for each of these attributes as well as other contributing factors, such as user satisfaction.^32^ The number of levels as well as the variety of attributes seems to depend on the patients’ specifics characteristics and the type of TR. In many included studies, the attributes’ levels lacked details and was often too imprecise^14,31,35,36,39,40^ to allow the choice of patients’ preferences. This could be explained by the fact that non DCE studies that provide less details on attributes’ levels because it was not their main objective. In this case, during focus groups and individual interviews before running the DCE studies, more details on attributes’ levels should be requested from patients about these attributes’ levels. Furthermore, attributes’ levels are characterized by their ascending or descending gradation and are generally well ordered.^17^ The challenge, however, is that these levels must be representative of what patients want. The treatment attributes and levels identified in the included studies will allow the elaboration of the rehabilitation scenario for future DCE studies. These scenarios will allow to establish the order of option’ preference according to the different levels of attributes.

####  Patients’ Satisfaction

 Patients’ satisfaction is an important aspect to consider in healthcare delivery models using TR^49^ because it could reinforce their adherence. For instance, the satisfaction for specific attributes of TR could potentially impact clinical outcomes through adherence. For patients without personal experience with TR, adherence may be influenced by factor such as their perception about TR. A positive perception could facilitate greater adherence contrary to a negative perception. According to satisfaction studies, some attributes such as “previous experience with technologies,”^14,37^ “ease of use,”^38^ “confidence or privacy,”^14,36^ “safety (emotional and data)”^30,36,39^ reinforced the list of the attributes identified. These studies provided some detailed information regarding patient attitudes toward the technology and the TR program. By understanding the characteristics that improve the patient satisfaction, healthcare providers can improve TR programs that provide accessible care.^50^

###  Study Implications 

 At the end of the period that included the search strategy, the two DCE studies found, were published during the past four years, and came from Europe and Australia, meaning that the topic of preferences in TR areas yet little been explored and could become of increasing importance in the field. To our knowledge, this is the first systematic review in the field of TR to identify the attributes and the levels the most important for patients in the choice of a TR treatment. Knowing the patients’ preference for identified attributes may potentially improve the outcome of the treatment, lead to a greater satisfaction,^7^ and a successful implementation of TR. In return, this could allow prioritizing healthcare resource allocation, as DCE studies provide a better understanding of the factors the most important to patients, and could be used to inform patient-centered TR implementation design.^17^ For future DCE research in the field of TR, this study represents a first step before the qualitative component that aim to determine the attributes underlying the choice of TR to support a DCE study design.

###  Strengths and Limitations 

 One major strength of this review is that we provided careful attention to screening and data extraction in the field of TR on the patient’s preference, adherence, satisfaction, and perception by following rigorously the PRISMA Statement. Second, this review allowed to identify several attributes and levels to develop an interview grid and topic guides to prepare individual interviews and focus groups. In addition, the stages of the development process for attributes were identified as well as the different methods used to estimate the relative importance for the attribute, which represents another strength of this study. Some limitations of this review must be emphasized. First, data extraction was conducted by one researcher as well as the quality appraisal of included studies. Second, no specific tools (except an Excel spreadsheet) to support data collection were used. This could be interpreted as a lack of methodological rigor and impact the quality of this review. Third, we decided not to exclude studies based on their quality since we wanted to be as exhaustive as possible and that future focus groups will select the most important attributes. Fourth, the lack of the protocol registration in the PROSPERO international database is a limitation to consider. In fact, the prospective registration of a protocol increases its transparency and reduces the likelihood of bias and unnecessary duplication of efforts.^51^ Fifth, although we employed a rigorous phased search, it is possible that we missed articles that were published in grey literature (ie, various website) or into a language other than English or French. Further articles^48^ could be published after the period that included the search strategy. We indirectly assessed the risk of bias of the included studies using the MMAT which is imperfect considering that this tool mostly evaluates the quality of mixed-methods studies and also has limitations that have been discussed elsewhere.^28^ Finally, the limited number of DCE studies included in the review did not allow to precisely order the preferences of patients for TR, which justify the need to conduct additional studies in this field. Despite these limitations, our finding generated a potential list of attributes to support the qualitative phase of the DCE design in line with this review’s objective.

## Conclusion

 To our knowledge, this is the first systematic review in the field of TR exploring which attributes are important for patients in their decision-making when choosing a TR treatment. This review could guide the development of interview grid for individual interviews and focus groups before to conduct a DCE study. By understanding the characteristics that enhance patients’ preferences, healthcare providers can create or improve TR programs that provide high-quality and accessible care. Future research is needed to determine the relative importance of the attributes.

## Acknowledgements

 The authors would like to thank the Research Chair in Telerehabilitation of the University of Sherbrooke for their financial support to conduct this study.

## Ethical issues

 This article does not contain any studies with human participants performed by any of the authors.

## Competing interests

 MT is a member of the Centre de recherche sur le vieillissement (CdRV), Sherbrooke, Québec. TGP is member of the FRQS-funded Centre de recherche de l’IUSMM and a fellow of the FRQS.

## Authors’ contributions

 LPC, TGP, and MT conceived and conducted the study. LPC wrote the manuscript and TGP and MT revised it critically.

## Funding

 This work was supported by the Research Chair in Telerehabilitation of the University of Sherbrooke.

## Supplementary files

Supplementary file 1. PRISMA Checklist.

Supplementary file 2. Bibliographic Search Strategy.

Supplementary file 3. Full-text Articles Excluded, With Reasons.
