# Supplementary material for: Attributes Underlying Patient Choice for Telerehabilitation Treatment: A Mixed-Methods Systematic Review to Support a Discrete Choice Experiment Study Design
Source: Int J Health Policy Manag. 2021 Nov 3;11(10):1991–2002. doi: 10.34172/ijhpm.2021.150 (PMC9808290; doi:10.34172/ijhpm.2021.150)
Supplement: Supplementary file 2 — Bibliographic Search Strategy. [file ijhpm-11-1991-s002.pdf]

Article title: Attributes Underlying Patient Choice for Telerehabilitation Treatment: A mixed-Methods Systematic Review to Support a Discrete Choice Experiment Study Design

Journal name: International Journal of Health Policy and Management (IJHPM)

Authors' information: Lucien P. Coulibaly[1,2]*, Thomas G. Poder[3,4], Michel Tousignant[1,2]

[1]Université de Sherbrooke, Sherbrooke, QC, Canada.

[2]Centre de Recherche sur le Vieillissement, Sherbrooke, QC, Canada.

[3]Département de Gestion, Évaluation et Politique de Santé, École de santé publique de l'Université de Montréal, Montréal, QC, Canada.

[4]Centre de recherche de l'Institut universitaire en santé mentale de Montréal, Montréal, QC, Canada.

(*Corresponding author: lucien.coulibaly@usherbrooke.ca)

Supplementary file 2. Bibliographic Search Strategy

BIBLIOGRAPHIC SEARCH STRATEGY ON PUBMED

((((((telerehabilitation[MeSH Terms]) OR (tele rehabilitation[MeSH Terms])) OR (telemedecine[MeSH Terms])) OR (telehealth[MeSH Terms])) OR (tele health[MeSH Terms])) OR (telecare[MeSH Terms]) AND ((fha[Filter]) AND (clinicaltrial[Filter] OR meta-analysis[Filter] OR randomizedcontrolledtrial[Filter] OR review[Filter] OR systematicreview[Filter]) AND (fft[Filter]) AND (humans[Filter]) AND (female[Filter] OR male[Filter]) AND (dentaljournals[Filter] OR medline[Filter] OR nursingjournals[Filter]) AND (english[Filter] OR french[Filter]) AND (2005:2020[pdat]))) AND (((((((Patient* preference*[MeSH Terms]) OR (Patient* satisfaction[MeSH Terms])) OR (Patient* perception[MeSH Terms])) OR (Patient*adherence[MeSH Terms])) OR (Patient* acceptance[MeSH Terms])) OR (discrete choice[MeSH Terms])) OR (treatment choice[MeSH Terms]) AND ((fha[Filter]) AND (clinicaltrial[Filter] OR meta-analysis[Filter] OR randomizedcontrolledtrial[Filter] OR review[Filter] OR systematicreview[Filter]) AND (fft[Filter]) AND (humans[Filter]) AND (female[Filter] OR male[Filter]) AND (dentaljournals[Filter] OR medline[Filter] OR nursingjournals[Filter]) AND (english[Filter] OR french[Filter]))),,"Abstract, Full text, Clinical Trial, Meta-Analysis, Randomized Controlled Trial, Review, Systematic Review, Humans, English, French, Female, Male, Dental journals, MEDLINE, Nursing journals","((((""telerehabilitation""[MeSH Terms] OR ""telerehabilitation""[MeSH Terms]) OR ""telemedicine""[MeSH Terms]) OR (""tele""[All Fields] AND ""health""[MeSH Terms])) AND (((((((""hasabstract""[All Fields] AND ((((""clinical trial""[Publication Type] OR ""meta analysis""[Publication Type]) OR ""randomized controlled trial""[Publication Type]) OR ""review""[Publication Type]) OR ""systematic review""[Filter])) AND ""loattrfull text""[Filter]) AND ""humans""[MeSH Terms]) AND (""female""[MeSH Terms] OR ""male""[MeSH Terms])) AND ((""jsubsetd""[All Fields] OR ""medline""[Filter]) OR ""jsubsetn""[All Fields])) AND (""english""[Language] OR ""french""[Language])) AND 2005/01/01:2020/12/31[Date - Publication])) AND (((((""patient*""[All Fields] AND ""personal satisfaction""[MeSH Terms]) OR (""patient*""[All Fields] AND ""perception""[MeSH Terms])) OR (((((((((""discrete""[All Fields] OR ""discretely""[All Fields]) OR ""discreteness""[All Fields]) OR ""discretization""[All Fields]) OR ""discretizations""[All Fields]) OR ""discretize""[All Fields]) OR ""discretized""[All Fields]) OR ""discretizes""[All Fields]) OR ""discretizing""[All Fields]) AND ""choice behavior""[MeSH Terms])) OR (((((((""therapeutics""[MeSH Terms] OR ""therapeutics""[All Fields]) OR ""treatments""[All Fields]) OR ""therapy""[MeSH Subheading]) OR ""therapy""[All Fields]) OR ""treatment""[All Fields]) OR ""treatment s""[All Fields]) AND ""choice behavior""[MeSH Terms]))

AND (((((((""hasabstract""[All Fields] AND ((((""clinical trial""[Publication Type] OR ""meta analysis""[Publication Type]) OR ""randomized controlled trial""[Publication Type]) OR ""review""[Publication Type]) OR ""systematic review""[Filter])) AND ""loattrfull text""[Filter]) AND ""humans""[MeSH Terms]) AND (""female""[MeSH Terms] OR ""male""[MeSH Terms])) AND ((""jsubsetd""[All Fields] OR ""medline""[Filter]) OR ""jsubsetn""[All Fields])) AND (""english""[Language] OR ""french""[Language])))",27,12:14:31

BIBLIOGRAPHIC SEARCH STRATEGY ON MEDLINE VIA EBSCO

#	Query	Limiters/Expanders	Results
S3	S1 AND S2	Limiters - Date of Publication: 20050101-20200731; Human Search modes - Boolean/Phrase	495
S2	AB Patient* preference* OR AB Patient* satisfaction OR AB Patient* perception OR AB Patient*adherence OR AB Patient* acceptance OR AB discrete choice experiment OR AB treatment choice	Search modes - Find all my search terms	301,693
S1	AB telerehabilitation OR AB tele rehabilitation OR AB telemedecine OR AB telehealth OR AB tele health OR AB telecare	Search modes - Find all my search terms	5,362

BIBLIOGRAPHIC SEARCH STRATEGY ON COCHRANE LIBRARY

ID Search Hits

#1 (telerehabilitation):ti,ab,kw OR (tele rehabilitation):ti,ab,kw OR (telemedecine):ti,ab,kw OR (telehealth):ti,ab,kw AND (telecare):ti,ab,kw with Publication Year from 2005 to 2020, with Cochrane Library publication date Between Jan 2005 and Jul 2020, in Trials (Word variations have been searched) 624

#2 (Patient* preference*):ti,ab,kw OR (Patient* satisfaction):ti,ab,kw OR (Patient* perception):ti,ab,kw OR (Patient*adherence):ti,ab,kw OR (Patient* acceptance):ti,ab,kw with Publication Year from 2005 to 2020, with Cochrane Library publication date Between Jan 2005 and Jul 2020, in Trials (Word variations have been searched) 73348

#3 ("discrete choice experiment"):ti,ab,kw OR (treatment choice):ti,ab,kw with Publication Year from 2005 to 2020, with Cochrane Library publication date Between Jan 2005 and Jul 2020, in Trials (Word variations have been searched) 11651

#4 #2 OR #3 with Publication Year from 2005 to 2020, with Cochrane Library publication date Between Jan 2005 and Jul 2020, in Trials (Word variations have been searched) 82858

#5 #1 AND #4 with Publication Year from 2005 to 2020, with Cochrane Library publication date Between Jan 2005 and Jan 2020, in Trials (Word variations have been searched) 139

BIBLIOGRAPHIC SEARCH STRATEGY ON SCIENCES DIRECT

#1 ("telerehabilitation" OR "tele-rehabilitation" OR "telehealth" OR “telecare”), 4087 document results

#2 ("patients preferences" OR "discrete choice experiment"), 22, 211 document results

#3 ("telerehabilitation" OR "tele-rehabilitation" OR "telehealth" OR “telecare”) AND ("patients preferences" OR "discrete choice experiment"), 233 document results

BIBLIOGRAPHIC SEARCH STRATEGY ON SCOPUS

#1 (TITLE-ABS-KEY(telerehabilitation) OR TITLE-ABS-KEY(tele-rehabilitation) OR TITLE-ABS-KEY(telemedecine) OR TITLE-ABS-KEY(telehealth) OR TITLE-ABS-KEY(tele health) OR TITLE-ABS-KEY(telecare)) AND DOCTYPE(ar OR re) AND RECENT(30), 237 document results

#2 (TITLE-ABS-KEY (patients AND preferences)

OR TITLE-ABS-KEY (patients AND satisfaction)

OR TITLE-ABS-KEY (patients AND perception)

OR TITLE-ABS-KEY (patients AND adherence)

OR TITLE-ABS-KEY (patients AND acceptance)

OR TITLE-ABS-KEY (discrete AND choice AND experiment)

OR TITLE-ABS-KEY (treatment AND choice))

AND DOCTYPE (ar OR re) AND RECENT (30), 3 026 document results

#1 AND #3 ((TITLE-ABS-KEY(telerehabilitation) OR TITLE-ABS-KEY(tele-rehabilitation) OR TITLE-ABS-KEY(telemedecine) OR TITLE-ABS-KEY(telehealth) OR TITLE-ABS-KEY(tele health) OR TITLE-ABS-KEY(telecare)) AND DOCTYPE(ar OR re) AND ORIG-LOAD-DATE aft 20200803) AND ((TITLE-ABS-KEY(patients preferences) OR TITLE-ABS-KEY(patients satisfaction) OR TITLE-ABS-KEY(patients perception) OR TITLE-ABS-KEY(patients adherence) OR TITLE-ABS-KEY(patients acceptance) OR TITLE-ABS-KEY(discrete choice experiment) OR TITLE-ABS-KEY(treatment choice)) AND DOCTYPE(ar OR re) AND ORIG-LOAD-DATE aft 20200803) AND RECENT(30), 33 document results
