# Supplementary material for: Attributes Underlying Patient Choice for Telerehabilitation Treatment: A Mixed-Methods Systematic Review to Support a Discrete Choice Experiment Study Design
Source: Int J Health Policy Manag. 2021 Nov 3;11(10):1991–2002. doi: 10.34172/ijhpm.2021.150 (PMC9808290; doi:10.34172/ijhpm.2021.150)
Supplement: Supplementary file 3 — Full-text Articles Excluded, With Reasons. [file ijhpm-11-1991-s003.pdf]

**Article title:** Attributes Underlying Patient Choice for Telerehabilitation Treatment: A mixed-Methods Systematic Review to Support a Discrete Choice Experiment Study Design

**Journal name:** International Journal of Health Policy and Management (IJHPM)

**Authors' information**: Lucien P. Coulibaly[1,2]*, Thomas G. Poder[3,4], Michel Tousignant[1,2]

[1]Université de Sherbrooke, Sherbrooke, QC, Canada.

[2]Centre de Recherche sur le Vieillissement, Sherbrooke, QC, Canada.

[3]Département de Gestion, Évaluation et Politique de Santé, École de santé publique de l'Université de Montréal, Montréal, QC, Canada.

[4]Centre de recherche de l'Institut universitaire en santé mentale de Montréal, Montréal, QC, Canada.

(*Corresponding author: lucien.coulibaly@usherbrooke.ca)

**Supplementary file 3.** Full-text Articles Excluded, With Reasons

| N° | References | Countries | Reasons of exclusion |
|---|---|---|---|
| 1 | Chumbler NR, Li X, Quigley P, et al. A randomized controlled trial on stroke telerehabilitation: the effects on falls self-efficacy and satisfaction with care. *Journal of telemedicine and telecare.* 2015;21(3):139-143. | U.S.A | Did not consider the preferences of patients |
| 2 | Cranen K, Drossaert CH, Brinkman ES, Braakman-Jansen AL, IJzerman MJ, Vollenbroek-Hutten MM. An exploration of chronic pain patients' perceptions of home telerehabilitation services. *Health expectations.* 2012;15(4):339-350. | Netherlands | Did not present the results of interest for TR' attributes and preferences |
| 3 | Grant LA, Rockwood T, Stennes L. Client satisfaction with telehealth services in home health care agencies. *Journal of Telemedicine and Telecare.* 2015;21(2):88-92. | U.S.A | Did not consider the preferences of patients |
| 4 | Chang J, Savage SJ, Waldman DM. Estimating willingness to pay for online health services with discrete-choice experiments. *Applied health economics and health policy.* 2017;15(4):491-500. | U.S.A | Study analyzed preferences but not for TR attributes |
| 5 | Odeh B, Kayyali R, Nabhani-Gebara S, Philip N, Robinson P, Wallace CR. Evaluation of a Telehealth Service for COPD and HF patients: Clinical outcome and patients' perceptions. *Journal of telemedicine and telecare.* 2015;21(5):292-297. | England | No DCE study |
| 6 | Knudsen LR, De Thurah A, Lomborg K. Experiences with telehealth follow-up in patients with rheumatoid | Denmark | No DCE study |

| | | | |
|---|---|---|---|
| | arthritis: A Qualitative Interview Study. *Arthritis Care & Research.* 2018;70(9):1366-1372. | | |
| 7 | Sanders C, Rogers A, Bowen R, et al. Exploring barriers to participation and adoption of telehealth and telecare within the Whole System Demonstrator trial: a qualitative study. *BMC health services research.* 2012;12(1):220. | England | No DCE study |
| 8 | Hwang R, Mandrusiak A, Morris NR, et al. Exploring patient experiences and perspectives of a heart failure telerehabilitation program: A mixed methods approach. *Heart & Lung.* 2017;46(4):320-327. | Australia | No DCE study |
| 9 | Pani D, Piga M, Barabino G, et al. Home tele-rehabilitation for rheumatic patients: impact and satisfaction of care analysis. *Journal of telemedicine and telecare.* 2017;23(2):292-300. | Italy | No DCE study |
| 10 | Young LB, Foster L, Silander A, Wakefield BJ. Home telehealth: patient satisfaction, program functions, and challenges for the care coordinator. *Journal of gerontological nursing.* 2011;37(11):38-46. | U.S.A | No DCE study |
| 11 | Fletcher B, Hinton L, McManus R, Rivero-Arias O. Patient preferences for management of high blood pressure in the UK: a discrete choice experiment. *British Journal of General Practice.* 2019;69(686):e629-e637. | England | Study analyzed preferences but not for TR attributes |
| 12 | Boyde M, Rankin J, Whitty JA, et al. Patient preferences for the delivery of cardiac rehabilitation. *Patient education and counseling.* 2018;101(12):2162-2169. | Australia | Study analyzed preferences but not for TR attributes |
| 13 | Onuma AE, Kelly EP, Chakedis J, et al. Patient preferences on the use of technology in cancer surveillance after curative surgery: A cross-sectional analysis. *Surgery.* 2019;165(4):782-788. | U.S.A | Study analyzed preferences but not for TR attributes |
| 14 | Moffet H, Tousignant M, Nadeau S, et al. Patient satisfaction with in-home telerehabilitation after total knee arthroplasty: results from a randomized controlled trial. *Telemedicine and e-Health.* 2017;23(2):80-87. | Canada | Did not consider the preferences of patients |
| 15 | Schein RM, Schmeler MR, Saptono A, Brienza D. Patient satisfaction with telerehabilitation assessments for wheeled mobility and seating. *Assistive Technology®.* 2010;22(4):215-222. | U.S.A | Did not consider the preferences of patients |
| 16 | Tousignant M, Boissy P, Corriveau H, Moffet H, Cabana F. In-home telerehabilitation for post-knee arthroplasty: A pilot study. *International Journal of Telerehabilitation.* 2009;1(1):9. | Canada | Did not present the results of interest for TR' attributes and preferences |

| | | | |
|---|---|---|---|
| 17 | Tousignant M, Boissy P, Moffet H, et al. Patients' satisfaction of healthcare services and perception with in-home telerehabilitation and physiotherapists' satisfaction toward technology for post-knee arthroplasty: an embedded study in a randomized trial. *Telemedicine and e-Health.* 2011;17(5):376-382. | Canada | Did not consider the preferences of patients |
| 18 | Polinski JM, Barker T, Gagliano N, Sussman A, Brennan TA, Shrank WH. Patients' satisfaction with and preference for telehealth visits. *Journal of general internal medicine.* 2016;31(3):269-275. | U.S.A | No DCE study |
| 19 | Rahimpour M, Lovell NH, Celler BG, McCormick J. Patients' perceptions of a home telecare system. *International journal of medical informatics.* 2008;77(7):486-498. | Australia | Did not present the results of interest for TR' attributes and preferences |
| 20 | Piron L, Turolla A, Tonin P, Piccione F, Lain L, Dam M. Satisfaction with care in post-stroke patients undergoing a telerehabilitation programme at home. *Journal of telemedicine and telecare.* 2008;14(5):257-260. | Italy | Did not consider the preferences of patients |
| 21 | Kairy D, Tousignant M, Leclerc N, Côté A-M, Levasseur M. The patient's perspective of in-home telerehabilitation physiotherapy services following total knee arthroplasty. *International journal of environmental research and public health.* 2013;10(9):3998-4011. | Canada | Did not present the results of interest for TR' attributes and preferences |
| 22 | Lilholt PH, Heiden S, Hejlesen OK. User satisfaction and experience with a telehealth system for the Danish TeleCare North Trial: a think-aloud study. *Studies in health technology and informatics.* 2014;205:900-904. | Denmark | Did not consider the preferences of patients |
| 23 | Becevic M, Boren S, Mutrux R, Shah Z, Banerjee S. User satisfaction with telehealth: study of patients, providers, and coordinators. *The health care manager.* 2015;34(4):337-349. | U.S.A | Did not present the results of interest for TR' attributes and preferences |
| 24 | Moffet H, Vincent C, Saey D, et al. Users' Perception and Readiness of the eChez-Soi In-Home Telerehabilitation Platform. Paper presented at: AAATE Conf.2015. | Canada | Did not present the results of interest for TR' attributes and preferences |
| 25 | Wentink MM, Prieto E, De Kloet A, Vliet Vlieland T, Meesters J. The patient perspective on the use of information and communication technologies and e-health in rehabilitation. *Disability and Rehabilitation: Assistive Technology.* 2018;13(7):620-625. | Netherlands | No DCE study |

| | | | |
|---|---|---|---|
| 26 | Demiris G, Speedie SM, Finkelstein S. Change of patients' perceptions of TeleHomeCare. *Telemedicine Journal and e-Health.* 2001;7(3):241-248. | U.S.A | Did not present the results of interest for TR' attributes and preferences |
| 27 | Whitten P, Mickus M. Home telecare for COPD/CHF patients: outcomes and perceptions. *Journal of telemedicine and telecare.* 2007;13(2):69-73. | U.S.A | Did not present the results of interest for TR' attributes and preferences |
| 28 | Corriveau H, Tousignant M, Gosselin S, Boissy P, Azevedo L, Gelderblom G. Patients satisfaction with an in-home telerehabilitation exercise program and physiotherapists' satisfaction toward technology for an acute stroke population: a pilot study. *Assistive Technology: from research to practice.* 2013;33:753-757. | Canada | Did not consider the preferences of patients |
| 29 | Sharma S, Ward EC, Burns C, Theodoros D, Russell T. Assessing dysphagia via telerehabilitation: patient perceptions and satisfaction. *International journal of speech-language pathology.* 2013;15(2):176-183. | Australia | No DCE study |
| 30 | Cottrell MA, Hill AJ, O'Leary SP, Raymer ME, Russell TG. Patients are willing to use telehealth for the multidisciplinary management of chronic musculoskeletal conditions: a cross-sectional survey. *Journal of telemedicine and telecare.* 2018;24(7):445-452. | Australia | No DCE study |
| 31 | Patients and physiotherapists satisfaction of in-home telerehabilittion for post-knee arthroplasty<br>M Tousignant, H Moffet, P Boissy, H Corriveau, F Cabana, E Marquis Physiotherapy (united kingdom)., 2011, 97, eS1246-eS1247 \| added to CENTRAL: 31 October 2015 \| 2015 | Canada | Did not consider the preferences of patients |
| 32 | Satisfaction assessment in poststroke patients exposed to a telerehabilitation trial, L Piron, P Tonin, M Dam, B Pizzoni, G Santarello, A Turolla, F, Piccione,Neurorehabilitation and neural repair, 2008, 22(5), 609 \| added to CENTRAL: 31 October 2009 \| 2009 Issue 4 | Italy | Did not consider the preferences of patients |
